# Systemic inflammatory biomarkers in gastroesophageal reflux disease: current evidence and future perspectives

**DOI:** 10.3389/fmed.2026.1893763

**Published:** 2026-08-07

**Authors:** Yu Liu, Lin Wang, Zhe Han, Zhongyu Wang, Wei Jia

**Affiliations:** 1 Hebei Medical University, Shijiazhuang, Hebei, China; 2 Department of Breast Surgery, Hengshui People's Hospital, Hengshui, Hebei, China; 3 Department of Nephrology, Baotou Central Hospital, Baotou, Inner Mongolia, China; 4 Gastrointestinal Disease Center, The First Hospital of Hebei Medical University, Shijiazhuang, Hebei, China; 5 Hebei University, Baoding, Hebei, China

**Keywords:** biomarkers, gastroesophageal reflux disease, inflammation, neutrophil-to-lymphocyte ratio, systemic immune-inflammation index

## Abstract

Gastroesophageal reflux disease (GERD) is a common gastrointestinal disorder that affects a large proportion of the population and is associated with impaired quality of life and increased healthcare utilization. Although GERD has traditionally been attributed to excessive reflux and dysfunction of the anti-reflux barrier, growing evidence indicates that inflammatory and immune-related processes also contribute to symptom development, mucosal injury, and disease progression. Accordingly, peripheral blood-derived inflammatory biomarkers, including the neutrophil-to-lymphocyte ratio (NLR), platelet-to-lymphocyte ratio (PLR), lymphocyte-to-monocyte ratio (LMR), and systemic immune-inflammation index (SII), have been investigated as simple and readily available indicators of systemic inflammation. Clinical studies have suggested that these hematological indices may be associated with the presence of GERD, endoscopic phenotypes, and disease severity, although reported values and cutoff thresholds remain inconsistent. In addition, chronic low-grade inflammation, impaired epithelial barrier function, obesity-associated metabolic inflammation, and alterations in the microbiome are increasingly recognized as interacting mechanisms that may influence both local esophageal injury and systemic inflammatory responses. However, published findings remain inconsistent, and the clinical utility of these biomarkers has yet to be clearly established. This review provides an overview of the inflammatory mechanisms involved in GERD and evaluates current evidence regarding the potential role of hematological inflammatory biomarkers in clinical practice. Their strengths, current limitations, and priorities for future research are also discussed.

## Introduction

GERD is a common chronic gastrointestinal disorder characterized by troublesome reflux symptoms and/or complications resulting from the reflux of gastric contents into the esophagus (1). The global prevalence of GERD has increased substantially over recent decades, particularly in industrialized countries, making it a major public health concern (2). Typical manifestations include heartburn and regurgitation, while extra-esophageal symptoms such as chronic cough, laryngitis, and chest pain may also occur. Persistent reflux can lead to erosive esophagitis, Barrett's esophagus, strictures, and esophageal adenocarcinoma.

The pathogenesis of GERD has traditionally been explained by mechanical and chemical factors, including transient lower esophageal sphincter relaxations, impaired esophageal clearance, delayed gastric emptying, hiatal hernia, and acid exposure (3). Although acid reflux remains central to disease development, growing evidence indicates that GERD is not solely a chemical injury-related disease (4). Recent studies have highlighted the potential involvement of inflammatory pathways, immune activation, epithelial barrier dysfunction, and microbiome alterations in the pathophysiology of GERD.

Chronic inflammatory and immune-mediated responses are increasingly recognized as relevant components of GERD pathophysiology (5). Exposure of the esophageal epithelium to refluxed gastric contents may activate inflammatory pathways involving cytokine and chemokine production, oxidative stress, and immune-cell recruitment (6). These processes may contribute not only to mucosal injury but also to symptom perception and disease chronicity.

In this context, hematological inflammatory biomarkers derived from routine blood examinations have emerged as promising indicators of systemic inflammatory status. Among these, the NLR has received particular attention because it reflects the balance between inflammatory activation and immune regulation (7). Other biomarkers, including PLR, LMR, and SII, have also been investigated in various inflammatory and neoplastic disorders (8), although their GERD-specific evidence remains limited.

Because these biomarkers are inexpensive, readily available, and reproducible, they may provide additional insights into GERD severity and phenotypes. This review aims to summarize current evidence regarding systemic inflammatory biomarkers in GERD, discuss their pathophysiological basis, and evaluate their potential clinical applications and future research directions.

## Literature search strategy

A structured literature search was conducted to identify studies evaluating systemic inflammatory biomarkers in GERD. The databases searched included PubMed, Embase, Web of Science, and the Cochrane Library.

The search terms included combinations of the following keywords and Medical Subject Headings terms: “gastroesophageal reflux disease,” “GERD,” “reflux esophagitis,” “erosive reflux disease,” “non-erosive reflux disease,” “Barrett's esophagus,” “neutrophil-to-lymphocyte ratio,” “NLR,” “platelet-to-lymphocyte ratio,” “PLR,” “lymphocyte-to-monocyte ratio,” “LMR,” “systemic immune-inflammation index,” “SII,” “C-reactive protein,” “cytokines,” and “inflammatory biomarkers.”

The search strategy was adapted for each database. Reference lists of relevant original articles and review articles were also manually screened to identify additional eligible studies. Because this article was designed as a narrative review rather than a systematic review, formal meta-analysis was not performed.

### Study selection criteria

Studies were considered eligible if they met the following criteria: (1) included patients diagnosed with GERD, reflux esophagitis, non-erosive reflux disease, erosive reflux disease, or Barrett's esophagus; (2) evaluated at least one systemic inflammatory biomarker, including NLR, PLR, LMR, SII, CRP, cytokines, chemokines, or related hematological inflammatory indices; (3) reported clinical, endoscopic, laboratory, or mechanistic findings relevant to GERD-associated inflammation; (4) were original clinical studies, mechanistic studies, systematic reviews, or high-quality narrative reviews relevant to the topic.

Exclusion criteria were as follows: (1) studies unrelated to GERD or reflux-related esophageal diseases; (2) studies focusing exclusively on non-GERD diseases without direct relevance to reflux-related inflammation; (3) case reports, conference abstracts, letters, and editorials without original data; (4) studies without accessible full text or sufficient biomarker-related information.

### Inflammatory mechanisms in GERD

#### Esophageal mucosal inflammation

Although acid exposure is a major initiating factor in GERD, inflammatory responses appear to play a pivotal role in mediating tissue injury and symptom development. Experimental and translational studies have shown that reflux exposure can induce the expression of pro-inflammatory mediators in esophageal epithelial and mucosal tissues, including IL-6, IL-8, IL-1β, TNF-α, and several chemokines (6, 9, 10). These cytokines promote recruitment of inflammatory cells and amplification of mucosal injury.

Histological studies of reflux esophagitis have demonstrated inflammatory-cell infiltration, including neutrophils and eosinophils, together with basal-cell hyperplasia and papillary elongation in affected esophageal mucosa (11). In addition, activation of nuclear factor kappa B (NF-κB) signaling pathways has been implicated in reflux-induced inflammatory responses (6). Persistent inflammatory activation may contribute to epithelial damage, ulcer formation, and Barrett's metaplasia.

Importantly, symptom severity in GERD is not always proportional to acid exposure alone. Some patients with non-erosive reflux disease (NERD) exhibit significant symptoms despite the absence of visible mucosal injury, suggesting that inflammatory sensitization and immune dysregulation may contribute to symptom generation (12).

#### Epithelial barrier dysfunction

Disruption of esophageal epithelial integrity is another important mechanism underlying GERD-associated inflammation. Tight junction proteins maintain mucosal barrier function and protect against chemical injury (13). Exposure to acid and bile salts may increase epithelial permeability and induce dilated intercellular spaces, facilitating penetration of refluxate into deeper mucosal layers.

Barrier dysfunction may enhance local inflammatory responses and activate sensory nerve endings, thereby contributing to visceral hypersensitivity (14). Increasing evidence suggests that inflammatory mediators and oxidative stress further aggravate epithelial damage, creating a vicious cycle between reflux injury and inflammation.

#### Immune activation and systemic inflammation

Local inflammatory activity in GERD may be accompanied by alterations in circulating inflammatory mediators; however, the extent to which local esophageal inflammation translates into measurable systemic inflammation remains uncertain. Neutrophils are among the earliest inflammatory cells recruited during tissue injury and may release proteolytic enzymes and reactive oxygen species (15). Conversely, lymphocyte suppression has been associated with chronic inflammatory stress (16, 17). Accordingly, peripheral blood cell-derived indices have been explored as indirect markers of inflammatory status in GERD, although their disease specificity and mechanistic relationship with esophageal inflammation remain unclear.

GERD-specific studies have reported alterations in circulating cytokines and mucosal inflammatory mediators, with more pronounced mucosal inflammatory activity observed in some patients with erosive reflux disease than in those with non-erosive disease or healthy controls (18, 19). These findings support an inflammatory component in GERD pathophysiology, although current evidence is stronger for local mucosal inflammation than for a consistent systemic inflammatory response (20, 21).

#### Obesity and metabolic inflammation

Obesity is a well-established risk factor for GERD (22, 23). In addition to increasing intra-abdominal pressure and promoting reflux, obesity is closely associated with chronic systemic inflammation. Adipose tissue secretes various pro-inflammatory cytokines and adipokines, including leptin, IL-6, and TNF-α (24). Leptin has also been shown to activate intracellular signaling pathways in esophageal-derived cells, although the clinical relevance of this finding to GERD remains uncertain (25).

Metabolic syndrome and insulin resistance have also been linked to GERD severity (26). Therefore, systemic inflammatory biomarkers may partly reflect obesity-related inflammatory pathways in GERD patients.

#### Microbiome and inflammatory responses

Emerging evidence suggests that alterations in the oral and esophageal microbiome may influence inflammatory pathways in GERD (27, 28). Microbial dysbiosis can affect epithelial integrity, immune activation, and cytokine production. Altered microbial communities may influence epithelial integrity and local immune signaling; however, causal relationships between specific microbial patterns, inflammation, and GERD progression have not yet been established.

Recent microbiome studies have demonstrated differences in bacterial composition between GERD patients and healthy controls (29). Although the precise mechanisms remain unclear, microbiota-related immune modulation may represent an important link between local mucosal injury and systemic inflammatory responses.

**Figure 1:**
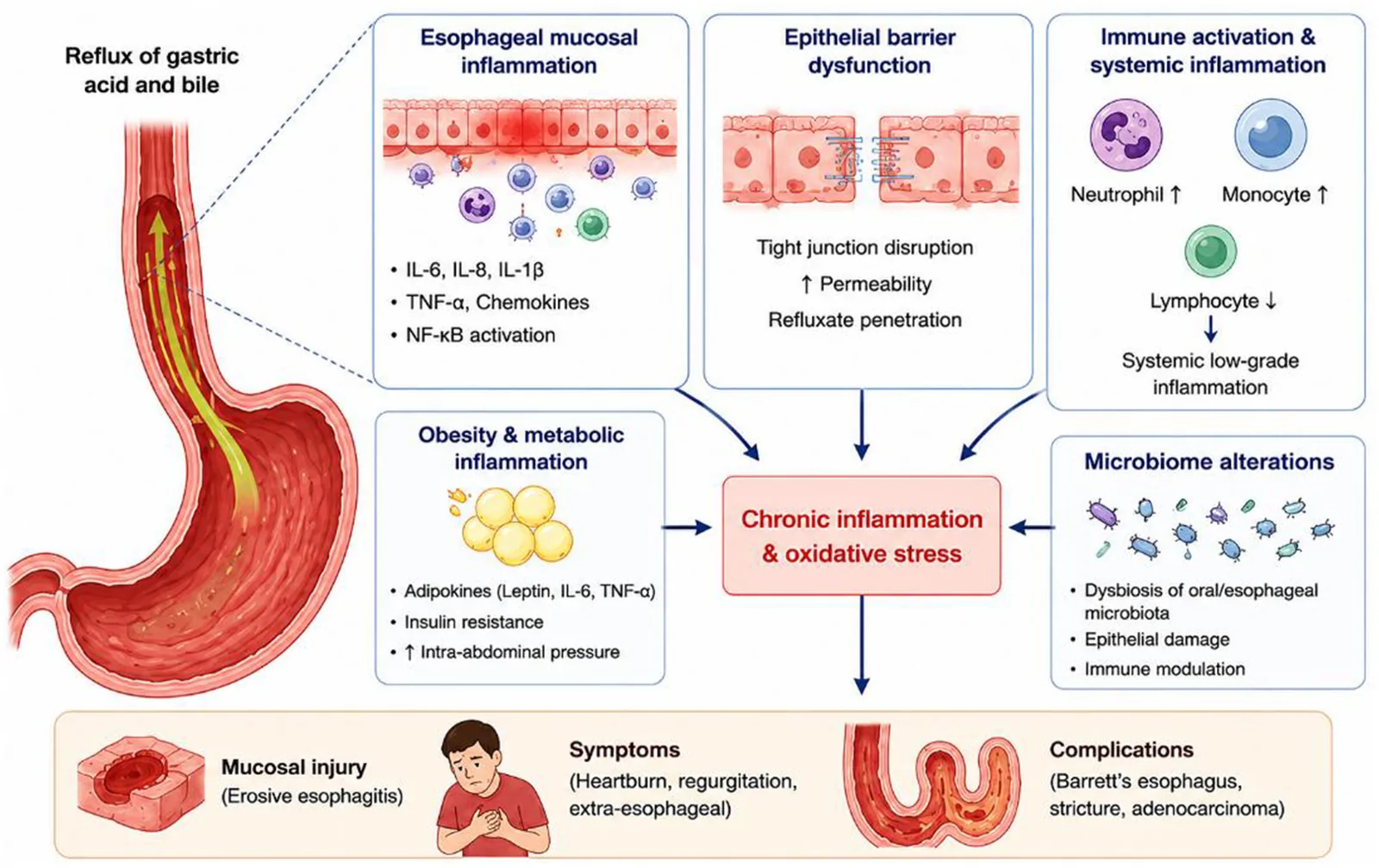
Inflammatory mechanisms in GERD.

Collectively, current evidence suggests that GERD involves interactions among reflux exposure, epithelial barrier dysfunction, mucosal immune activation, obesity-related metabolic factors, and microbiome alterations. The relative contribution of these mechanisms is likely to differ among GERD phenotypes and remains incompletely defined (30, 31) (Figure 1).

### Hematological inflammatory biomarkers in GERD

In the following sections, GERD-specific studies are prioritized when discussing clinical associations, whereas evidence from other disease areas is used primarily to explain the biological basis of each biomarker.

### Neutrophil-to-lymphocyte ratio

The NLR is one of the most widely studied inflammatory biomarkers in clinical medicine (7, 32). It is calculated by dividing the absolute neutrophil count by the absolute lymphocyte count obtained from routine blood tests. NLR reflects the balance between innate inflammatory activation and adaptive immune regulation (33).

Although NLR is a non-specific marker of systemic inflammation, its relevance to GERD should be interpreted primarily on the basis of GERD-specific evidence. A GERD-focused study suggested that NLR may be associated with the endoscopic severity of reflux esophagitis (34). Therefore, in this review, non-GERD-specific evidence is used only to explain the biological rationale of inflammatory indices, whereas conclusions regarding clinical utility are based mainly on GERD-related studies.

A retrospective study reported that NLR was associated with the severity of reflux esophagitis (34). Furthermore, patients with erosive reflux disease (ERD) may demonstrate higher NLR levels than those with NERD (34). A recent retrospective cohort study suggested that NLR may be associated with progression from Barrett's esophagus to esophageal adenocarcinoma (35). These findings suggest that NLR may correlate with the degree of mucosal inflammation.

Some investigators have proposed that elevated NLR may be associated with severe reflux symptoms, increased endoscopic severity, or prolonged disease duration (34). However, the reported values and diagnostic performance vary among studies, and no universally accepted GERD-specific cutoff has been established.

The potential clinical advantages of NLR include its low cost, wide availability, and ease of calculation. Nevertheless, NLR lacks disease specificity and may be influenced by multiple confounding factors such as infection, smoking, obesity, metabolic disorders, and medication use (36). Consequently, NLR should be interpreted cautiously and in conjunction with clinical findings.

### Platelet-to-lymphocyte ratio

Platelets play important roles in inflammatory and immune-mediated processes in addition to hemostasis. Activated platelets can release cytokines, chemokines, and growth factors that contribute to inflammatory responses (37, 38).

The PLR has therefore emerged as another inflammatory biomarker of interest. Some studies have suggested elevated PLR values in GERD patients, particularly in those with erosive disease, although the available evidence remains limited. PLR may reflect both inflammatory activation and thrombo-inflammatory interactions (39).

Compared with NLR, PLR has been less extensively studied in GERD. Current evidence suggests that PLR may have adjunctive value, but its independent clinical utility and optimal cutoff remain uncertain.

### Lymphocyte-to-monocyte ratio

The LMR is another hematological marker associated with systemic inflammatory status. Monocytes participate in chronic inflammation and tissue remodeling through cytokine production and differentiation into macrophages (40).

Reduced LMR has been associated with adverse outcomes in inflammatory and malignant diseases. LMR has been evaluated in a study that included GERD as a comparator group, but its specific role in GERD remains inconclusive (39). Further research is required to determine whether LMR provides additional value in assessing GERD severity or inflammatory burden.

### Systemic immune-inflammation index

The SII, calculated using neutrophil, platelet, and lymphocyte counts, has recently gained attention as a comprehensive marker of inflammatory and immune status (41).

Because SII incorporates three hematological parameters, it may better reflect complex inflammatory responses than individual indices alone. However, GERD-specific evidence regarding SII remains limited, and further studies are needed to clarify its clinical significance.

Despite its potential advantages, evidence regarding SII in GERD remains limited. Large-scale prospective studies are needed before routine clinical implementation can be recommended.

### C-reactive protein and other biomarkers

C-reactive protein (CRP) is a widely recognized marker of systemic inflammation and has been investigated in GERD patients (42). Some studies have reported elevated CRP levels in patients with erosive esophagitis or obesity-related GERD, although CRP values may also be influenced by obesity and other metabolic or inflammatory conditions (43).

Other inflammatory mediators, including cytokines, oxidative stress markers, and adhesion molecules, have also been explored. However, most of these biomarkers are costly, less accessible, and not routinely used in clinical practice.

Compared with specialized inflammatory markers, hematological indices offer practical advantages because they are inexpensive and readily obtainable from standard blood examinations.

**Figure 2:**
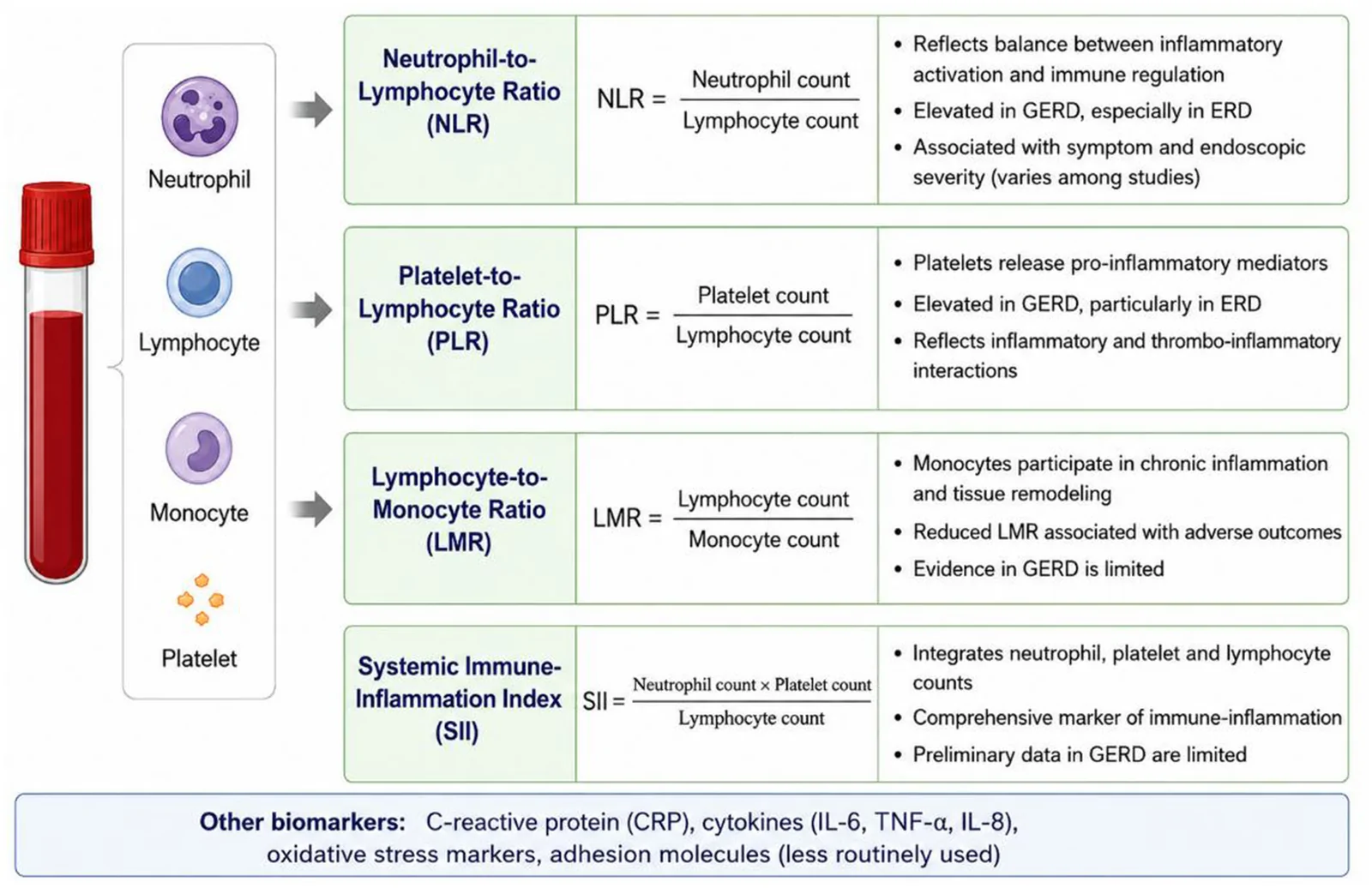
Hematological inflammatory biomarkers in GERD.

These hematological indices may collectively reflect the balance between inflammatory activation and immune regulation in GERD and therefore represent promising adjunctive biomarkers for evaluating disease-related inflammatory burden (Figure 2). Reported values, phenotype-related associations, and the current status of proposed cutoff values are summarized in Table 1.

**Table 1:** Summary of systemic inflammatory biomarkers discussed in GERD.

Biomarker	Biological relevance/function	Quantitative findings in GERD	Cutoff value	Associated phenotype	References
NLR	Reflects the balance between neutrophil-mediated inflammation and lymphocyte-mediated immune regulation	Higher values have been reported in GERD and reflux esophagitis; NLR may increase with endoscopic severity	No universally accepted GERD-specific cutoff	GERD, ERD, reflux esophagitis	(32–36)
PLR	Reflects platelet-related inflammatory activity and lymphocyte-mediated immune status	Elevated values have been reported in some GERD patients, particularly those with erosive disease, but evidence remains limited	Not established	Possible association with ERD	(37–39)
LMR	Reflects the balance between lymphocyte-mediated immunity and monocyte-related inflammation	Limited and inconclusive data	Not established	Not clearly established	(39, 40)
SII	Integrates neutrophil, platelet, and lymphocyte counts	GERD-specific quantitative evidence remains limited	Not established	Not clearly established	(41)
CRP	Reflects systemic acute-phase inflammatory activity	Elevated levels have been reported in some patients with erosive or obesity-related GERD	Not established	Erosive and obesity-related GERD	(42, 43)

### Relationship between inflammatory biomarkers and GERD phenotypes

#### Erosive and non-erosive disease

GERD encompasses heterogeneous clinical phenotypes, including erosive reflux disease and non-erosive reflux disease (44). Several studies have suggested that inflammatory biomarker levels are generally higher in ERD than in NERD, possibly reflecting greater mucosal injury and inflammatory activation (45).

However, NERD patients may also exhibit elevated inflammatory indices despite the absence of visible endoscopic lesions. This observation supports the hypothesis that immune-mediated hypersensitivity and microscopic inflammation may contribute to symptom generation in NERD.

#### Barrett's esophagus

Barrett's esophagus represents a premalignant condition arising from chronic reflux-related injury. Persistent inflammation is considered an important factor in Barrett's metaplasia and progression toward dysplasia and adenocarcinoma.

Persistent reflux-related inflammation has been implicated in Barrett's metaplasia and its progression, with evidence of an inflammatory gradient across Barrett's mucosa (45). Further investigation is necessary to determine whether these markers could aid risk stratification or surveillance strategies.

#### Refractory GERD

Proton pump inhibitors (PPIs) are the mainstay of GERD treatment; however, a subset of patients continues to experience symptoms despite adequate therapy. Persistent inflammation, visceral hypersensitivity, and non-acid reflux may contribute to refractory disease (46).

Systemic inflammatory biomarkers may potentially help identify patients with ongoing inflammatory activation despite acid suppression. Nevertheless, current evidence is insufficient to support routine clinical use in this setting.

#### Obesity-related GERD

Obesity-related GERD represents a clinically important phenotype associated with chronic metabolic inflammation. Elevated inflammatory biomarkers in obese GERD patients may reflect interactions between reflux injury and adipose tissue-derived cytokines.

Understanding these inflammatory mechanisms may have therapeutic implications, particularly regarding lifestyle intervention and weight reduction.

**Figure 3:**
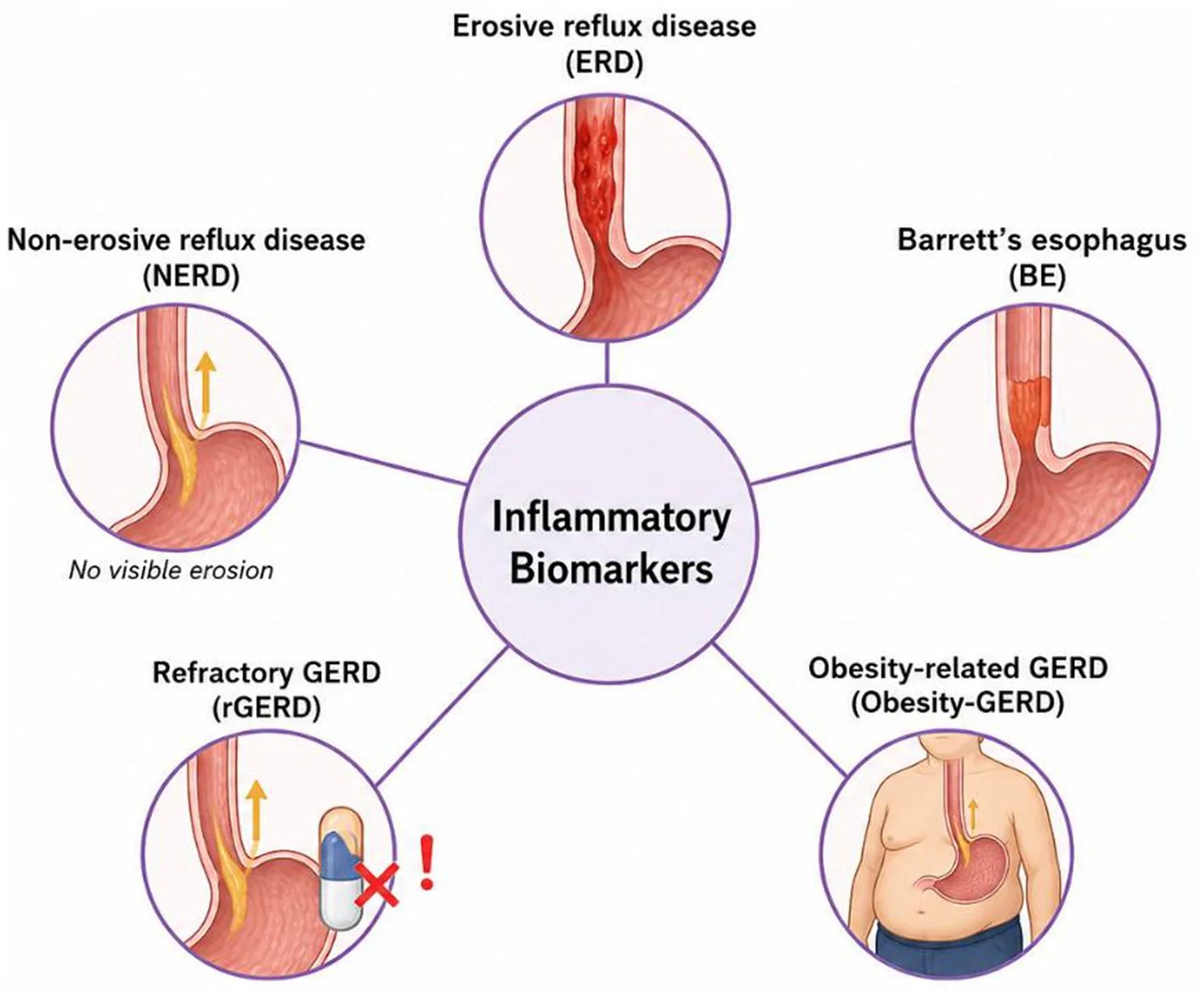
Relationship between inflammatory biomarkers and GERD phenotypes.

The heterogeneous distribution of inflammatory biomarkers among different GERD phenotypes further supports the concept that GERD represents a spectrum of disorders with distinct underlying inflammatory and pathophysiological mechanisms (Figure 3).

### Clinical implications of inflammatory biomarkers in GERD

#### Diagnostic assistance

Although hematological inflammatory indices are not diagnostic for GERD, GERD-related inflammatory activation has been supported by studies showing altered circulating cytokines and mucosal inflammatory mediators (20). Elevated inflammatory indices could potentially support the presence of inflammatory activity in symptomatic individuals.

However, these biomarkers are non-specific and cannot replace endoscopy, pH monitoring, or established diagnostic criteria. Their primary value may lie in complementing clinical assessment rather than establishing diagnosis independently.

#### Assessment of disease severity

Limited evidence suggests that systemic inflammatory indices may be associated with inflammatory burden in GERD; however, direct evidence linking NLR, PLR, or SII to endoscopic severity remains insufficient and requires further validation.

#### Prognostic and therapeutic potential

Inflammatory biomarkers may also have prognostic significance. Monitoring changes in inflammatory indices during treatment could potentially reflect therapeutic response or disease progression.

In addition, increasing recognition of inflammatory mechanisms in GERD may encourage exploration of novel anti-inflammatory or microbiome-targeted therapies in selected patients.

#### Limitations of current evidence

Despite growing interest in inflammatory biomarkers in GERD, several important limitations should be acknowledged.

First, most available studies are retrospective and single-center in design, limiting generalizability. Sample sizes are often relatively small, and study populations are heterogeneous.

Second, there is substantial variability in biomarker cutoff values, laboratory methods, and patient selection criteria. Standardized methodologies are lacking.

Third, inflammatory indices are affected by numerous confounding factors, including infection, smoking, obesity, diabetes, cardiovascular disease, medications, and psychological stress. These factors may reduce specificity for GERD-related inflammation.

Fourth, causal relationships remain unclear. Elevated inflammatory biomarkers may represent secondary epiphenomena rather than direct contributors to GERD pathogenesis.

Finally, current evidence regarding microbiome-inflammation interactions in GERD remains preliminary. Further mechanistic studies are required to clarify these complex relationships.

#### Future perspectives

Future research should focus on large-scale prospective studies to validate the diagnostic and prognostic value of systemic inflammatory biomarkers in GERD. Standardization of cutoff values and study methodologies will be essential.

Integration of inflammatory biomarkers with endoscopic findings, pH monitoring, symptom assessment, and microbiome profiling may improve phenotypic characterization of GERD. Multidimensional approaches could facilitate more personalized management strategies.

In addition, growing interest in the microbiome-immune axis may provide new insights into disease mechanisms. Further investigation into the interactions among microbial dysbiosis, epithelial barrier dysfunction, immune activation, and systemic inflammation may help identify novel therapeutic targets.

Artificial intelligence-based predictive models integrating clinical and inflammatory data may also enhance risk stratification in the future.

**Figure 4:**
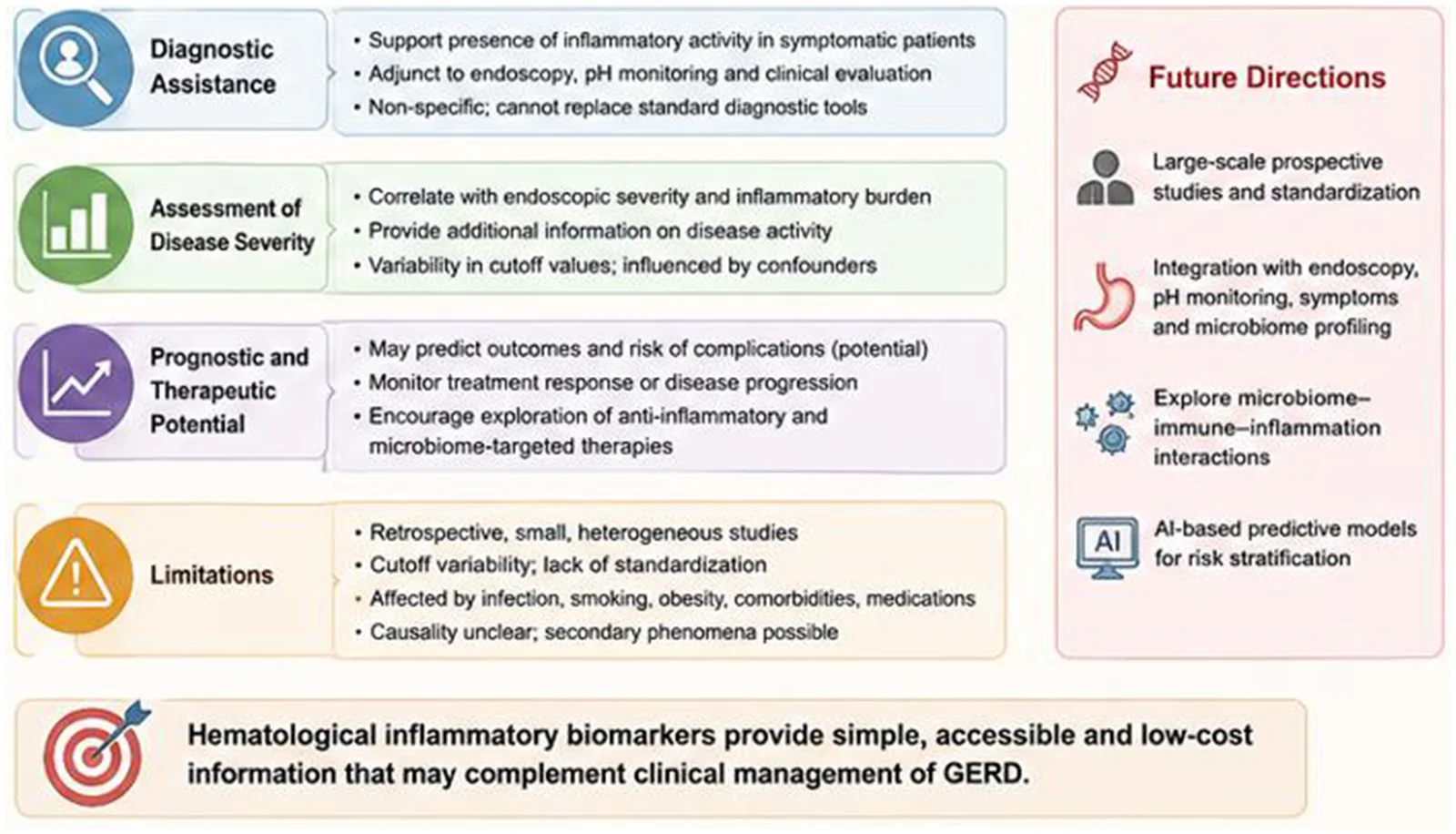
Clinical implications of inflammatory biomarkers in GERD.

With further validation and standardization, inflammatory biomarkers may become integrated into multidimensional diagnostic and prognostic models, thereby contributing to more personalized management approaches for GERD in the future (Figure 4).

## Conclusion

Increasing evidence suggests that gastroesophageal reflux disease involves not only chemical injury caused by gastric reflux but also chronic inflammatory and immune-mediated mechanisms. Hematological inflammatory biomarkers, particularly NLR, PLR, and SII, have emerged as promising indicators of systemic inflammatory status in GERD.

Current studies demonstrate associations between elevated inflammatory indices and GERD severity, erosive phenotypes, and chronic inflammatory activity. However, available evidence remains heterogeneous and limited by methodological weaknesses.

Although these biomarkers are unlikely to replace conventional diagnostic tools, they may provide useful adjunctive information because of their simplicity, accessibility, and low cost. Future well-designed prospective studies integrating inflammatory biomarkers, microbiome analysis, and clinical phenotyping may further improve understanding and management of GERD.
